# Several genetic polymorphisms interact with overweight/obesity to influence serum lipid levels

**DOI:** 10.1186/1475-2840-11-123

**Published:** 2012-10-08

**Authors:** Rui-Xing Yin, Dong-Feng Wu, Lin Miao, Lynn Htet Htet Aung, Xiao-Li Cao, Ting-Ting Yan, Xing-Jiang Long, Wan-Ying Liu, Lin Zhang, Meng Li

**Affiliations:** 1 Department of Cardiology, Institute of Cardiovascular Diseases, the First Affiliated Hospital, Guangxi Medical University, 22 Shuangyong Road, Nanning, Guangxi, 530021, People's Republic of China; 2 Department of Cardiology, The Third Affiliated Hospital, Guangxi Medical University, Nanning, Guangxi, People's Republic of China; 3 Department of Cardiology, The People's Hospital of Guilin, Guilin, Guangxi, People's Republic of China; 4 Department of Internal Medicine, Worker's Hospital of Guangxi Liuzhou Iron and Steel (Group) Company, Liuzhou, Guangxi, People's Republic of China

**Keywords:** Lipid, Apolipoprotein, Genetic polymorphism, Overweight, Obesity, Interaction

## Abstract

**Background:**

Information about the interactions of single nucleotide polymorphisms (SNPs) and overweight/obesity on serum lipid profiles is still scarce. The present study was undertaken to detect ten SNPs and their interactions with overweight/obesity on serum lipid levels.

**Methods:**

A total of 978 normal weight and 751 overweight/obese subjects of Bai Ku Yao were randomly selected from our previous stratified randomized cluster samples. Normal weight, overweight and obesity were defined as a body mass index (BMI) < 24, 24–28, and > 28 kg/m^2^; respectively. Serum total cholesterol (TC), triglyceride (TG), high-density lipoprotein cholesterol (HDL-C), low-density lipoprotein cholesterol (LDL-C), apolipoprotein (Apo) A1 and ApoB levels were measured. Genotyping of ATP-binding cassette transporter A1 (ABCA-1) V825I, acyl-CoA:cholesterol acyltransferase-1 (ACAT-1) rs1044925, low density lipoprotein receptor (LDL-R) *Ava*II, hepatic lipase gene (LIPC) -250G>A, endothelial lipase gene (LIPG) 584C>T, methylenetetrahydrofolate reductase (MTHFR) 677C>T, the E3 ubiquitin ligase myosin regulatory light chain-interacting protein (MYLIP) rs3757354, proprotein convertase subtilisin-like kexin type 9 (PCSK9) E670G, peroxisome proliferator-activated receptor delta (PPARD) +294T>C, and Scavenger receptor class B type 1 (SCARB1) rs5888 was performed by polymerase chain reaction and restriction fragment length polymorphism combined with gel electrophoresis, and then confirmed by direct sequencing. The interactions were detected by factorial design covariance analysis.

**Results:**

The genotypic and allelic frequencies of LIPC and PCSK9 were different between normal weight and overweight/obese subjects, the genotypic frequency of LIPG and allelic frequency of MYLIP were also different between normal weight and overweight/obese subjects (*P* < 0.05-0.001). The levels of TC, ApoA1 (ABCA-1); TC, LDL-C, ApoA1, ApoB and ApoA1/ApoB (LIPC); TG, HDL-C, and ApoA1 (LIPG); TC, HDL-C, LDL-C, ApoA1 and ApoB (MTHFR); HDL-C and ApoA1 (MYLIP) in normal weight subjects were different among the genotypes (*P* < 0.01-0.001). The levels of LDL-C, ApoB and ApoA1/ApoB (ABCA-1); HDL-C, ApoA1, ApoB and ApoA1/ApoB (LIPC); TC, HDL-C, ApoA1 and ApoB (LIPG); TC, TG, HDL-C, LDL-C, ApoA1 and ApoB (MTHFR); TC, TG and ApoB (MYLIP); TG (PCSK9); TG, ApoA1 and ApoB (PPARD); and TC, HDL-C, LDL-C, ApoA1 and ApoB (SCARB1) in overweight/obese subjects were different among the genotypes (*P* < 0.01-0.001). The SNPs of ABCA-1 (LDL-C and ApoA1/ApoB); LIPC (TC, LDL-C, ApoA1 and ApoB); LIPG (ApoB); MTHFR (TC, TG and LDL-C); MYLIP (TC and TG); PCSK9 (TG, HDL-C, ApoB and ApoA1/ApoB); PPARD (TG and ApoA1/ApoB); and SCARB1 (TG, ApoA1 and ApoB) interacted with overweight/obesity to influence serum lipid levels (*P* < 0.05-0.001).

**Conclusions:**

The differences in serum lipid levels between normal weight and overweight/obese subjects might partly result from different genetic polymorphisms and the interactions between several SNPs and overweight/obesity.

## Introduction

Dyslipidemia such as elevated levels of total cholesterol (TC) [1], triglyceride (TG) [2], low-density lipoprotein cholesterol (LDL-C) [3], and apolipoprotein (Apo) B [4], together with decreased levels of ApoA1 [4] and high-density lipoprotein cholesterol (HDL-C) [5] has become one of the most urgent public health problems in many countries because of its high prevalence and a causal relationship with serious medical condition such as coronary artery disease (CAD), hypertension and stroke [6]. It is well known that dyslipidemia is a complex trait caused by multiple environmental and genetic factors and their interactions [7-12]. The link between overweight/obesity and dyslipidemia has been well documented [13-17]. Obesity is a specific phenotype that may be affected by genetic and environmental factors, involving excessive caloric intake, unhealthy lifestyle, insufficient physical activity, social and economic forces, as well as metabolic and endocrine abnormalities. The increase in body fat, especially the intra-abdominal adipose tissue is a major contributor to the development of dyslipidemia, insulin resistance, and hypertension and is associated with chronic diseases such as type 2 diabetes, CAD, metabolic syndrome, stroke, sleep disorders, osteoarthritis, and increased incidence of certain forms of cancer [17]. The prevalence of obesity has dramatically increased during recent years in all parts of the world [18]. According to the World Health Organization (WHO), more than 400 million adults were obese in 2005, and it is estimated that more than 700 million adults will be obese by 2015 [19]. Moreover, the rates of increase and the overall prevalence of obesity vary greatly across ethnic groups [20]. Among Americans, data from the National Health and Nutrition Examination Survey (NHANES) conducted in 2007–2008 showed that adults of 32.8% of non-Hispanic whites, 44.1% of non-Hispanic blacks, and 39.3% of Mexican-Americans were either overweight or obese [21]. The prevalence of overweight and obesity in Chinese was 24.1% and 2.8% in men and 26.1% and 5.0% in women; respectively. The prevalence of central obesity was 16.1% in men and 37.6% in women. The prevalences of overweight, obesity, and central obesity were higher among residents in northern China compared with their counterparts in southern China and among those in urban areas compared with those in rural areas [22]. Obesity has become a major clinical and public health problem that threatens to overwhelm already extended healthcare services in many countries. Genetic influences on lipid traits have been suggested by numerous studies. Recent large-scale genome-wide association studies in multiple populations have identified more than 95 loci associated with serum lipid levels [23]. Common variants at these loci together explain < 10% of variation in each lipid trait [24-26]. Rare variants with large individual effects may also contribute to the heritability of lipid traits [26]. But the results of these association studies conducted with blood lipid traits are inconsistent in diverse racial/ethnic groups. A major reason for inconsistency among studies may be different environmental modifiers that interact with genes to influence serum lipid levels.

China is a multiethnic country with 56 ethnic groups. Han nationality is the largest ethnic group, and Yao nationality is the eleventh largest minority among the 55 minority groups according to the population size. Bai Ku Yao (White-trouser Yao), an isolated subgroup of the Yao minority, is named so because all of men wear white knee-length knickerbockers. The population size is about 30 000. Because of isolation from the other ethnic groups, the special customs and cultures including their clothing, intra-ethnic marriages, dietary patterns, and corn wine and rum intakes are still completely preserved to the present day. Thus, this ethnic subgroup is thought to share the same ethnic ancestry and to possess a homogeneous genetic background. Bai Ku Yao has become a useful subgroup for population genetic studies. In previous epidemiological studies, we found that the prevalence of dyslipidemia was lower in normal weight than in overweight/obese subjects [7-9]. We hypothesized that the differences in serum lipid levels between normal weight and overweight/obese subjects might partly result from different interactions of some single nucleotide polymorphisms (SNPs) and overweight/obesity in this population. Therefore, the purpose of this study was to detect ten SNPs in different genes and evaluate their interactions with overweight/obesity on serum lipid levels in the Guangxi Bai Ku Yao population. The SNPs were selected according to the previous findings of genome-wide association studies [23-26] and bioinformatics functional assessment. Computational analysis of ten SNPs ascribed potential functional characteristics to each variant allele. In addition, the ten SNPs selected for genotyping also based on the frequency of Beijing Han population from the Human Genome Project Database. The heterozygosity values were higher than 10% for the minor allele frequency. These SNPs have been associated with serum lipid profiles in the Bai Ku Yao population [27-35].

## Methods

### Study population

The study population consisted of 1729 unrelated participants of Bai Ku Yao who reside in Lihu and Baxu villages in Nandan County, Guangxi Zhuang Autonomous Region, People's Republic of China. They were randomly selected from our previous stratified randomized cluster samples [7-9]. The age of the subjects ranged from 15 to 86 years, with an average age of 41.38 ± 14.71 years. There were 978 normal weight subjects (490 males and 488 females) and 751 overweight/obese subjects (378 men and 373 women). All of the subjects were rural agricultural workers. The subjects had no evidence of diseases related to atherosclerosis, CAD and diabetes. The participants were not taking medications known to affect serum lipid levels (lipid-lowering drugs such as statins or fibrates, beta-blockers, diuretics, hormones, or contraceptive drugs). The protocol was approved by the Ethics Committee of the First Affiliated Hospital, Guangxi Medical University. Written informed consent was obtained from each participant.

### Epidemiological survey

The survey was done according to standardized methods [7-9]. Questionnaires were administered to assess demographic information, socioeconomic status, lifestyle factors, and medical and medication history. Blood pressure was measured three times by a well trained physician with the use of a standard mercury sphygmomanometer while subjects were seated and had rested for 5 min. Systolic and diastolic blood pressure values were the mean of three measurements. Systolic blood pressure was determined by the first Korotkoff sound, and diastolic blood pressure by the fifth Korotkoff sound. Pulse pressure was calculated as the systolic minus the diastolic blood pressure. Weight was measured with a beam balance and height with a fixed stadiometer. Subjects were measured without shoes and in a minimum of clothing. Body mass index (BMI) was calculated as weight in kilograms divided by the square of height in meters. Waist circumference was measured at the umbilicus.

### Biochemical measurements

Fasting venous blood samples of 5 mL were obtained from all subjects. A part of the sample (2 mL) was collected into glass tubes and used to determine serum lipid levels. Another part of the sample (3 mL) was transferred to tubes with anticoagulate solution and used to extract deoxyribonucleic acid (DNA). The levels of TC, TG, HDL-C, and LDL-C in samples were determined by enzymatic methods. Serum ApoA1 and ApoB levels were detected by the immunoturbidimetric immunoassay. All determinations were performed with an autoanalyzer (Type 7170A; Hitachi Ltd., Tokyo, Japan) in our Clinical Science Experiment Center [7-9].

### Genetic analyses

Genomic DNA was extracted from the peripheral blood leukocytes by the phenol-chloroform method as our previous reports [27-35]. Genotyping of ATP-binding cassette transporter A1 (ABCA-1) V825I (rs2066715), acyl-CoA:cholesterol acyltransferase-1 (ACAT-1) rs1044925, low density lipoprotein receptor (LDL-R) *Ava*II, hepatic lipase gene (LIPC) -250G>A (rs2070895), endothelial lipase gene (LIPG) 584C>T (rs2000813), methylenetetrahydrofolate reductase (MTHFR) 677C>T (rs1801133), the E3 ubiquitin ligase myosin regulatory light chain-interacting protein (MYLIP, also known as IDOL) rs3757354, proprotein convertase subtilisin-like kexin type 9 (PCSK9) E670G (rs505151), peroxisome proliferator-activated receptor delta (PPARD) +294T>C (rs2016520) and Scavenger receptor class B type 1 (SCARB1) rs5888 was performed using polymerase chain reaction and restriction fragment length polymorphism (PCR-RFLP). The sequences of the forward and reverse primers and restriction ezyme used for the genotyping of ten SNPs are list in Table 1. The thermocycling protocol, the approach to electrophoresis, and the procedures for quality control have been described previously [27-35]. Genotypes were scored by an experienced reader blinded to epidemiological data and serum lipid levels.

**Table 1:** The sequences of forward (F) and reverse (R) primers and restriction enzymes for genotyping of the ten SNPs

**SNP**	**Primer sequence**	**Restriction enzyme**	**PCR product**	**Allele**
ABCA-1 V825I	F: 5′-GGTAGCCCACCACTCTCCCCTATAAAG-3′	*Tag*I	525 bp	G
(rs2066715)	R: 5′-ATCAGCTGCCTGTCCTTGGACTA-3′			A
ACAT-1	F: 5′-TATATTAAGGGGATCAGAAGT-3′	*Rsa*I	389 bp	A
(rs1044925)	R: 5′-CCACCTAAAAACATACTACC-3′			C
LDL-R *Ava*II	F: 5′-GTCATCTTCCTTGCTGCCTGTTTAG-3′	*Ava*II	228 bp	A-
	R: 5′-GTTTCCACAAGGAGGTTTCAAGGTT-3′			A+
LIPC -250G>A	F: 5′-GGCAAGGGCATCTTTGCTTC-3′	*Dra*I	411-bp	G
(rs2070895)	R: 5′-GGTCGATTTACAGAAGTGCTTC-3′			A
LIPG 584C>T	F: 5′-CATGAGCTGAGATTGTTGTCAGTGC-3′	*Nde*I	254 bp	C
(rs2000813)	R: 5′-CAGTCAACCACAACTACATTGGCGTCTTTCTCTCAT-3′			T
MTHFR 677C>T	F: 5′-CAAAGGCCACCCCGAAGC-3′	*Hinf*I	254 bp	C
(rs1801133)	R: 5′-AGGACGGTGCGGTGAGAGTG-3′			T
MYLIP	F: 5′-ACAGAGCAAAAGACCCTGTCTC-3′	*Hae*III	387 bp	G
(rs3757354)	R: 5′-AAAGAACTGTGTGTGGGAGGAT-3′			T
PCSK9 E670G	F: 5′-CACGGTTGTGTCCCAAATGG-3′	*Eam* 1104I	440 bp	A
(rs505151)	R: 5′-GAGAGGGACAAGTCGGAACC-3′			G
PPARD +294T>C	F: 5′-CATGGTATAGCACTGCAGGAA-3′	*Bsl*I	269 bp	T
(rs2016520)	R: 5′-CTTCCTCCTGTGGCTGCTC-3′			C
SCARB1	F: 5′-CCTTGTTTCTCTCCCATCCTCACTTCCTCGACGC-3′	*Hin*I1	218 bp	C
(rs5888)	R: 5′-CACCACCCCAGCCCACAGCAGC-3′			T

### DNA sequencing

Fifty-eight samples (each genotype in two; respectively) detected by the PCR-RFLP were also confirmed by direct sequencing. The PCR products were purified by low melting point gel electrophoresis and phenol extraction, and then the DNA sequences were analyzed in Shanghai Sangon Biological Engineering Technology & Services Co., Ltd., People's Republic of China.

### Diagnostic criteria

The normal values of serum TC, TG, HDL-C, LDL-C, ApoA1, ApoB levels and the ratio of ApoA1 to ApoB in our Clinical Science Experiment Center were 3.10-5.17, 0.56-1.70, 1.16-1.42, 2.70-3.10 mmol/L, 1.20-1.60, 0.80-1.05 g/L, and 1.00-2.50; respectively. The individuals with TC > 5.17 mmol/L and/or TG > 1.70 mmol/L were defined as hyperlipidemic [7-9]. The diagnostic criteria of overweight and obesity were according to the Cooperative Meta-analysis Group of China Obesity Task Force. Normal weight, overweight and obesity were defined as a BMI < 24, 24–28, and > 28 kg/m^2^; respectively [14,36].

### Statistical analysis

Data are presented as means ± SD for continuous variables and as frequencies or percentages for categorical variables. Chi square tests were used to compare the differences in percentages and to assess Hardy-Weinberg expectations. Differences in mean values were assessed using analysis of covariance (ANCOVA) and unpaired *t* tests. Potential confounding factors were sex, age, education level, physical activity, blood pressure, alcohol consumption, and cigarette smoking. All significant associations were further corrected for multiple tests by a permutation test. The permutation test was conducted by changing the orders of dependant variable randomly against the genotypes (under the null hypothesis - no association between dependant variable and haplotypes). This process was repeated 1000 times. The *P* values of 1000 permutations were sorted in a descending manner. If the observed *P* value is less than or equal to the 950^th^*P* value, the association was considered statistically significant. The allelic and genotypic frequencies were calculated from the observed genotypic counts. The interactions of ten SNPs and overweight/obesity on serum lipid levels were assessed by using a factorial design covariance analysis after controlling for potential confounders. Multiple linear regression was used to ascertain the correlation between genotypes (ABCA-1: GG = 1, GA = 2, AA = 3; ACAT-1: AA = 1, AC = 2, CC = 3; LDL-R: A-A- = 1, A-A+ = 2, A+A+ = 3; LIPC: GG = 1, GA = 2, AA = 3; LIPG: CC = 1, CT = 2, TT = 3; MTHFR: CC = 1, CT = 2, TT = 3; MYLIP: AA = 1, AG = 2, GG = 3; PCSK9: AA = 1, AG = 2; PPARD: TT = 1, TC = 2, CC = 3; and SCARB1: CC = 1, CT = 2, TT = 3) or alleles (the minor allele noncarrier = 1, the minor allele carrier = 2) and serum lipid parameters in the combined population of normal weight and overweight/obese subjects, normal weight subjects, and overweight/obese subjects; respectively.

## Results

### General characteristics

Table 2 shows the general characteristics of the participants. The levels of education, weight, BMI, waist circumference, systolic blood pressure, diastolic blood pressure, serum TC, TG, LDL-C, ApoA1, ApoB, and the percentages of subjects who consumed alcohol were higher in overweight/obese than in normal weight subjects (*P* < 0.05-0.001), whereas the levels of serum HDL-C, the ratio of ApoA1 to ApoB, and the percentages of subjects who smoked cigarettes were lower in overweight/obese than in normal weight subjects (*P* < 0.01 for all). There were no significant differences in the levels of mean age, height, pulse pressure, and the ratio of male to female between the overweight/obese and normal weight subjects (*P* > 0.05 for all).

**Table 2:** The general characteristics and serum lipid levels between the subjects with normal weight and overweight/obesity

**Characteristics**	**Normal weight**	**Overweight/obesity**	***t *(χ**^**2**^**)**	***P***
Number	978	751	–	–
Male/female	490/488	378/373	0.009	0.924
Age, years	41.48±16.21	41.25±12.50	0.332	0.740
Education level, years	3.71±3.89	4.71±4.49	−4.170	0.000
Height, cm	153.73±7.57	154.29±8.49	−1.461	0.144
Weight, kg	50.28±6.20	63.19±8.86	−34.042	0.000
Body mass index, kg/m^2^	21.23±1.68	26.48±2.59	−48.285	0.000
Waist circumference, cm	70.58±6.56	82.90±7.39	−30.766	0.000
Alcohol consumption, *n* (%)	373 (38.1)	362 (48.2)	23.034	0.000
Cigarette smoking, *n* (%)	305 (31.2)	178 (23.7)	15.036	0.001
Systolic blood pressure, mmHg	119.69±17.40	125.84±17.64	−7.242	0.000
Diastolic blood pressure, mmHg	75.11±9.98	80.57±11.13	−10.563	0.000
Pulse pressure, mmHg	44.60±12.87	45.36±12.11	−1.251	0.211
Total cholesterol, mmol/L	4.46±0.94	5.01±1.05	−11.325	0.000
Triglyceride, mmol/L	1.21±1.02	1.74±1.50	−8.837	0.000
HDL-C, mmol/L	1.80±0.47	1.73±0.41	3.099	0.002
LDL-C, mmol/L	2.52±0.73	2.96±0.85	−11.180	0.000
Apolipoprotein (Apo) A1, g/L	1.37±0.31	1.40±0.27	−2.009	0.045
ApoB, g/L	0.84±0.22	0.98±0.24	−12.466	0.000
ApoA1/ApoB	1.75±0.70	1.53±0.58	7.256	0.000

HDL-C, high-density lipoprotein cholesterol; LDL-C, low-density lipoprotein cholesterol.

### Electrophoresis and genotypes

**Figure 1:**
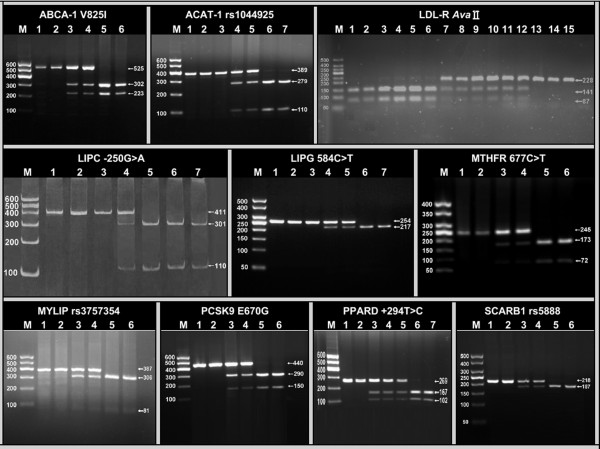
**Genotyping of ten SNPs by polymerase chain reaction and restriction fragment length polymorphism.** ABCA-1, ATP-binding cassette transporter A1; ACAT-1, acyl-CoA:cholesterol acyltransferase-1; LDL-R, low density lipoprotein receptor; LIPC, hepatic lipase gene; LIPG, endothelial lipase gene; MTHFR, methylenetetrahydrofolate reductase; MYLIP, the E3 ubiquitin ligase myosin regulatory light chain-interacting protein. PCSK9, proprotein convertase subtilisin-like kexin type 9; PPARD, peroxisome proliferator-activated receptor delta; SCARB1, Scavenger receptor class B type 1.

The PCR products of ABCA-1, ACAT-1, LDL-R, LIPC, LIPG, MTHFR, MYLIP, PCSK9, PPARD, and SCARB1 SNPs were 525-, 389-, 228-, 411-, 254-, 254-, 387-, 440-, 269- and 218-bp nucleotide sequences; respectively. The genotypes identified were named according to the presence or absence of the enzyme restriction sites (Figure 1). Lane M is 50- or 100-bp marker ladder. The genotypes of the ten SNPs are as follows: ABCA1, GG (lanes 1 and 2, 525-bp), GA (lanes 3 and 4, 525-, 302- and 223-bp), and AA genotypes (lanes 5 and 6, 302- and 223-bp); ACAT-1, AA (lanes 1-3, 389-bp), AC (lanes 4 and 5, 389-, 279- and 110-bp), and CC genotypes (lanes 6 and 7, 279- and 110-bp); LDL-R, A+A+ (lanes 1-6, 141- and 87-bp), A-A+ (lanes 7-12, 228-, 141- and 87-bp), and A-A- genotypes (lanes 13-15, 228-bp); LIPC, GG (lanes 1-3, 411-bp), GA (lane 4, 411-, 301- and 110-bp), and AA genotypes (lanes 5-7, 301- and 110-bp); LIPG, PCR product of the sample (lane 1, 254-bp), CC (lanes 2 and 3, 254-bp), CT (lanes 4 and 5, 254-, 217- and 37-bp), and TT genotypes (lanes 6 and 7, 217- and 37-bp); MTHFR, CC (lanes 1 and 2, 245-bp), CT (lane 3 and 4, 245-, 173- and 72-bp), and TT genotypes (lanes 5 and 6, 173- and 72-bp); MYLIP, AA (lanes 1 and 2, 387-bp), AG (lanes 3 and 4, 387-, 306- and 81-bp), and GG genotypes (lanes 5 and 6, 306- and 81-bp); PCSK9, PCR products of the samples (lanes 1 and 2, 440-bp), AG (lanes 3 and 4, 440-, 290- and 150-bp), and AA genotypes (lanes 5 and 6, 290- and 150-bp); PPARD, TT (lanes 1 and 2, 269-bp), TC (lanes 3-5, 269-, 167- and 102-bp), and CC genotypes (lanes 6 and 7, 167- and 102-bp); and SCARB1, TT (lanes 1 and 2, 218-bp), CT (lanes 3 and 4, 218-, 187- and 31-bp), and CC genotypes (lanes 5 and 6, 187- and 31-bp). The 37-bp fragment of LIPG and 31-bp fragment of SCARB1 were invisible in the gel owing to their fast migration speed. The GG homozygous of the PCSK9 E670G was not detected in our study population.

### Nucleotide sequences

**Figure 2:**
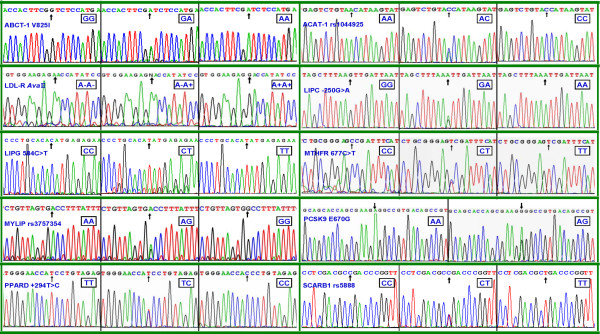
**The parts of the nucleotide sequence of ten SNPs by direct sequencing.** ABCA-1, ATP-binding cassette transporter A1; ACAT-1, acyl-CoA:cholesterol acyltransferase-1; LDL-R, low density lipoprotein receptor; LIPC, hepatic lipase gene; LIPG, endothelial lipase gene; MTHFR, methylenetetrahydrofolate reductase; MYLIP, the E3 ubiquitin ligase myosin regulatory light chain-interacting protein. PCSK9, proprotein convertase subtilisin-like kexin type 9; PPARD, peroxisome proliferator-activated receptor delta; SCARB1, Scavenger receptor class B type 1.

The genotypes detected by PCR-RFLP were also confirmed by direct sequencing (Figure 2).

### Genotypic and allelic frequencies

The genotypic and allelic frequencies of the SNPs between normal weight and overweight/obese subjects are summarized in Table 3. The genotypic distribution of ten SNPs was in Hardy-Weinberg equilibrium (*P* > 0.05 for all). The genotypic and allelic frequencies of LIPC and PCSK9 were different between normal weight and overweight/obese subjects, the overweight/obese subjects had higher LIPC -250A and PCSK9 670A allele frequencies than normal weight subjects (*P* < 0.05-0.001). The genotypic frequency of LIPG and allelic frequency of MYLIP were also different between normal weight and overweight/obese subjects (*P* < 0.05 for each). There were no significant differences in the genotypic and allelic frequencies of the remaining SNPs between normal weight and overweight/obese subjects (*P* < 0.05 for all). The GG homozygous of the PCSK9 E670G was not detected in our study population.

**Table 3:** The genotypic and allelic frequencies between the subjects with normal weight and overweight/obesity [n (%)]

**SNP**	**Genotype (Allele)**	**Normal weight (n = 978)**	**Overweight/obesity (n = 751)**	**χ**^**2**^	***P***
ABCA-1 V825I	GG	326 (33.3)	269 (35.8)		
(rs2066715)	GA	480 (49.1)	334 (44.5)		
	AA	172 (17.6)	148 (19.7)	3.708	0.157
	G	1132 (57.9)	872 (58.1)		
	A	824 (42.1)	630 (41.9)	0.012	0.914
ACAT-1	AA	662 (67.7)	527 (70.2)		
(rs1044925)	AC	279 (28.5)	205 (27.3)		
	CC	37 (3.8)	19 (2.5)	2.671	0.263
	A	1603 (82.0)	1259 (83.8)		
	C	353 (18.0)	243 (16.2)	2.080	0.149
LDL-R *Ava*II	A-A-	527 (53.9)	389 (51.8)		
	A-A+	371 (37.9)	295 (39.3)		
	A+A+	80 (8.2)	67 (8.9)	0.824	0.662
	A-	1425 (72.9)	1073 (71.4)		
	A+	531 (27.1)	429 (28.6)	0.848	0.357
LIPC -250G>A	GG	480 (49.1)	233 (31.0)		
(rs2070895)	GA	425 (43.5)	432 (57.5)		
	AA	73 (7.5)	86 (11.5)	57.882	0.000
	G	1385 (70.8)	898 (59.8)		
	A	571 (29.2)	604 (40.2)	45.999	0.000
LIPG 584C>T	CC	454 (46.4)	308 (41.0)		
(rs2000813)	CT	477 (48.8)	412 (54.9)		
	TT	47 (4.8)	31 (4.1)	6.314	0.043
	C	1385 (70.8)	1028 (68.4)		
	T	571 (29.2)	474 (31.6)	2.255	0.133
MTHFR 677C>T	CC	471 (48.2)	354 (47.1)		
(rs1801133)	CT	441 (45.1)	341 (45.4)		
	TT	66 (6.7)	56 (7.5)	0.404	0.817
	C	1383 (70.7)	1049 (69.8)		
	T	573 (29.3)	453 (30.2)	0.305	0.581
MYLIP	AA	230 (23.5)	148 (19.7)		
(rs3757354)	AG	477 (48.8)	363 (48.3)		
	GG	271 (27.7)	240 (32.0)	5.431	0.066
	A	937 (47.9)	659 (43.9)		
	G	1019 (52.1)	843 (56.1)	5.550	0.018
PCSK9 E670G	AA	916 (93.7)	721 (96.0)		
(rs505151)	AG	62 (6.3)	30 (4.0)		
	GG	0	0	4.636	0.031
	A	1894 (96.8)	1472 (98.0)		
	G	62 (3.2)	30 (2.0)	4.509	0.034
PPARD +294T>C	TT	559 (57.2)	396 (52.7)		
(rs2016520)	TC	354 (36.2)	312 (41.5)		
	CC	65 (6.6)	43 (5.7)	5.239	0.073
	T	1472 (75.3)	1104 (73.5)		
	C	484 (24.7)	398 (26.5)	1.375	0.241
SCARB1	CC	548 (56.0)	417 (55.5)		
(rs5888)	CT	390 (39.9)	311 (41.4)		
	TT	40 (4.1)	23 (3.1)	1.497	0.473
	C	1486 (76.0)	1145 (76.2)		
	T	470 (24.0)	357 (23.8)	0.032	0.859

SNP, single nucleotide polymorphism; ABCA-1, ATP-binding cassette transporter A1; ACAT-1, acyl-CoA:cholesterol acyltransferase-1; LDL-R, low density lipoprotein receptor; LIPC, hepatic lipase gene; LIPG, endothelial lipase gene; MTHFR, methylenetetrahydrofolate reductase; MYLIP, the E3 ubiquitin ligase myosin regulatory light chain-interacting protein; PCSK9, proprotein convertase subtilisin-like kexin type 9; PPARD, peroxisome proliferator-activated receptor delta; SCARB1, Scavenger receptor class B type 1.

### Genotypes and serum lipid levels

**Figure 3:**
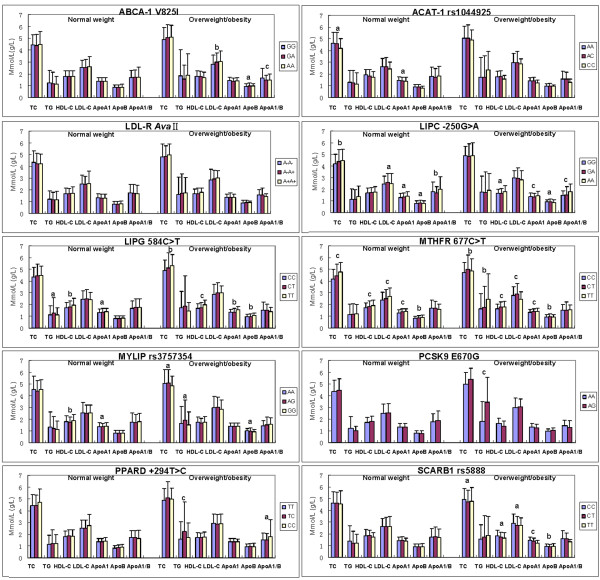
**The genotypes of ten SNPs and serum lipid levels between normal weight and overweight/obese subjects.** TC, total cholesterol; TG, triglyceride; HDL-C, high-density lipoprotein cholesterol; LDL-C, low-density lipoprotein cholesterol; ApoA1, apolipoprotein A1; ApoB, apolipoprotein B; ApoA1/B, the ratio of apolipoprotein A1 to apolipoprotein B; ABCA-1, ATP-binding cassette transporter A1; ACAT-1, acyl-CoA:cholesterol acyltransferase-1; LDL-R, low density lipoprotein receptor; LIPC, hepatic lipase gene; LIPG, endothelial lipase gene; MTHFR, methylenetetrahydrofolate reductase; MYLIP, the E3 ubiquitin ligase myosin regulatory light chain-interacting protein; PCSK9, proprotein convertase subtilisin-like kexin type 9; PPARD, peroxisome proliferator-activated receptor delta; SCARB1, Scavenger receptor class B type 1; ^a^*P* < 0.01, ^b^*P* < 0.005, and ^c^*P* < 0.001 (after permutation correction).

The association of genotypes and serum lipid parameters between normal weight and overweight/obese subjects is shown in Figure 3. The levels of TC, ApoA1 (ABCA-1); TC, LDL-C, ApoA1, ApoB and ApoA1/ApoB (LIPC); TG, HDL-C, and ApoA1 (LIPG); TC, HDL-C, LDL-C, ApoA1 and ApoB (MTHFR); HDL-C and ApoA1 (MYLIP) in normal weight subjects were different among the genotypes (*P* < 0.01-0.001).

The levels of LDL-C, ApoB and ApoA1/ApoB (ABCA-1); HDL-C, ApoA1, ApoB and ApoA1/ApoB (LIPC); TC, HDL-C, ApoA1 and ApoB (LIPG); TC, TG, HDL-C, LDL-C, ApoA1 and ApoB (MTHFR); TC, TG and ApoB (MYLIP); TG (PCSK9); TG and ApoA1 and ApoB (PPARD); and TC, HDL-C, LDL-C, ApoA1 and ApoB (SCARB1) in overweight/obese subjects were different among the genotypes (*P* < 0.01-0.001).

### Interactions of the SNPs and overweight/obesity on serum lipid levels

The interactions of ten SNPs and overweight/obesity on serum lipid levels are given in Table 4. The SNPs of ABCA-1 (LDL-C and ApoA1/ApoB); LIPC (TC, LDL-C, ApoA1 and ApoB); LIPG (ApoB); MTHFR (TC, TG and LDL-C); MYLIP (TC and TG); PCSK9 (TG, HDL-C, ApoB and ApoA1/ApoB); PPARD (TG and ApoA1/ApoB); and SCARB1 (TG, ApoA1 and ApoB) were shown interactions with overweight/obesity to influence serum lipid levels (*P* < 0.05-0.001).

**Table 4:** Interactions of several SNPs and overweight/obesity on serum lipid levels

**SNP**	**Genotype**	**TC (mmol/L)**	**TG (mmol/L)**	**HDL-C (mmol/L)**	**LDL-C (mmol/L)**	**ApoA1 (g/L)**	**ApoB (g/L)**	**ApoA1/ ApoB**
ABCA-1 V825I	GG	–	–	–	–	–	–	–
(rs2066715)	GA	–	–	–	↑	–	–	↓
	AA	–	–	–	↑	–	–	↓
	*F*	1.473	2.937	1.717	3.687	1.913	2.941	6.485
	*P*	0.230	0.053	0.180	0.005^c^	0.148	0.053	0.000^c^
ACAT-1 rs1044925	AA	–	–	–	–	–	–	–
(rs1044925)	AC	–	–	–	–	–	–	–
	CC	–	–	–	–	–	–	–
	*F*	0.475	1.457	0.341	0.320	1.635	1.071	1.944
	*P*	0.622	0.233	0.711	0.726	0.195	0.343	0.143
LDL-R *Ava*II	A-A-	–	–	–	–	–	–	–
	A-A+	–	–	–	–	–	–	–
	A+A+	–	–	–	–	–	–	–
	*F*	1.681	0.242	0.553	0.857	1.377	0.430	0.462
	*P*	0.186	0.785	0.576	0.425	0.253	0.650	0.630
LIPC -250G>A	GG	↑	–	–	↑	–	↑	–
(rs2070895)	GA	–	–	–	–	–	↑	–
	AA	–	–	–	–	↑	–	–
	*F*	3.733	0.266	1.584	3.345	3.292	4.138	2.116
	*P*	0.005^c^	0.766	0.205	0.007^c^	0.007^c^	0.003^c^	0.121
LIPG 584C>T	CC	–	–	–	–	–	–	–
(rs2000813)	CT	–	–	–	–	–	–	–
	TT	–	–	–	–	–	↑	–
	*F*	1.286	0.210	0.357	1.452	2.195	3.616	1.812
	*P*	0.277	0.810	0.700	0.234	0.112	0.005^c^	0.164
MTHFR 677C>T	CC	–	–	–	↑	–	–	–
(rs1801133)	CT	↑	–	–	–	–	–	–
	TT	–	↑	–	↓	–	–	–
	*F*	3.110	3.463	0.627	9.236	0.058	2.684	0.675
	*P*	0.009^c^	0.006^c^	0.534	0.000^c^	0.944	0.069	0.509
MYLIP	AA	–	–	–	–	–	–	–
(rs3757354)	AG	↑	↑	–	–	–	–	–
	GG	–	–	–	–	–	–	–
	*F*	6.864	4.325	1.818	2.495	0.684	1.895	1.388
	*P*	0.000^c^	0.003^c^	0.307	0.083	0.505	0.151	0.250
PCSK9 E670G	AA	–	–	–	–	–	–	–
(rs505151)	AG	–	↑	↓	–	–	↑	↓
	*F*	0.980	25.662	7.831	0.002	1.371	6.319	4.357
	*P*	0.322	0.000^c^	0.001^c^	0.968	0.242	0.002^c^	0.007^c^
PPARD +294T>C	TT	–	–	–	–	–	–	–
(rs2016520)	TC	–	↑	–	–	–	–	–
	CC	–	–	–	–	–	–	↑
	*F*	1.832	5.737	1.235	1.087	0.171	1.362	3.985
	*P*	0.160	0.001^c^	0.291	0.337	0.843	0.257	0.004^c^
SCARB1	CC	–	–	–	–	–	↑	–
(rs5888)	CT	–	–	–	–	↓	–	–
	TT	–	↑	–	–	↓	–	–
	*F*	1.809	4.396	1.720	2.211	3.226	3.118	0.051
	*P*	0.164	0.002^c^	0.179	0.110	0.008^c^	0.009^c^	0.778

SNP, single nucleotide polymorphism; TC, total cholesterol; TG, triglyceride; HDL-C, high-density lipoprotein cholesterol; LDL-C, low-density lipoprotein cholesterol; ApoA1, apolipoprotein A1; ApoB, apolipoprotein B; ApoA1/ApoB, the ratio of apolipoprotein A1 to apolipoprotein B; ABCA-1, ATP-binding cassette transporter A1; ACAT-1, acyl-CoA:cholesterol acyltransferase-1; LDL-R, low density lipoprotein receptor; LIPC, hepatic lipase gene; LIPG, endothelial lipase gene; MTHFR, methylenetetrahydrofolate reductase; MYLIP, the E3 ubiquitin ligase myosin regulatory light chain-interacting protein; PCSK9, proprotein convertase subtilisin-like kexin type 9; PPARD, peroxisome proliferator-activated receptor delta; SCARB1, Scavenger receptor class B type 1; “C” is the *P-*value after permutation correction. ↑: genotype and overweight/obesity interactions to increase serum lipid levels; ↓: genotype and overweight/obesity interactions to decrease serum lipid levels; –: no interaction of genotypes and overweight/obesity on serum lipid levels.

### Correlation between genotypes or alleles and serum lipid parameters

The results of multiple linear regression analysis between genotypes or alleles and serum lipid parameters are shown in Tables 5, 6, 7. Serum lipid levels were also associated with the genotypes or alleles of several SNPs in the combined population of normal weight and overweight/obese subjects (Table 5), normal weight subjects (Table 6), and overweight/obese subjects (Table 7) ; respectively (*P* < 0.05-0.001).

**Table 5:** Correlation between genotypes or alleles and serum lipid levels in the total population

**Lipid**	**Genotype**/**allele**	**Unstandardized coefficient**	**Std. error**	**Standardized coefficient**	***t***	***P***
TC	ACAT-1 rs1044925 genotype	−0.099	0.043	−0.053	−2.287	0.022
	LIPC -250G>A allele	0.101	0.046	0.050	2.170	0.030
	LIPG 584C>T genotype	0.149	0.043	0.079	3.503	0.000
	LIPG 584C>T allele	0.169	0.049	0.077	3.425	0.001
	MTHFR 677C>T genotype	0.218	0.037	0.133	5.854	0.000
	MTHFR 677C>T allele	0.269	0.046	0.133	5.841	0.000
	PPARD +294T>C allele	0.147	0.050	0.068	2.963	0.003
TG	MTHFR 677C>T genotype	0.146	0.054	0.062	2.701	0.007
	MYLIP rs3757354 genotype	−0.102	0.042	−0.057	−2.456	0.014
	PPARD +294T>C allele	0.321	0.086	0.087	3.732	0.000
	SCARB1 rs5888 genotype	−0.041	0.023	−0.049	−2.039	0.042
HDL-C	ACAT-1 rs1044925 genotype	−0.057	0.022	−0.062	−2.643	0.008
	ACAT-1 rs1044925 allele	−0.051	0.025	−0.048	−2.024	0.043
	LIPC -250G>A genotype	0.038	0.016	0.055	2.366	0.018
	LIPG 584C>T genotype	0.086	0.018	0.108	4.667	0.000
	LIPG 584C>T allele	0.080	0.021	0.087	3.762	0.000
	MTHFR 677C>T genotype	0.097	0.017	0.132	5.718	0.000
	MTHFR 677C>T allele	0.123	0.021	0.136	5.868	0.000
	MYLIP rs3757354 genotype	0.036	0.015	0.058	2.465	0.014
LDL-C	ABCA-1 V825I genotype	0.075	0.025	0.068	2.954	0.003
	MTHFR 677C>T genotype	0.075	0.029	0.059	2.562	0.010
	MTHFR 677C>T allele	0.120	0.036	0.077	3.334	0.001
ApoA1	ACAT-1 rs1044925 genotype	−0.042	0.013	−0.074	−3.143	0.002
	ACAT-1 rs1044925 allele	−0.042	0.016	−0.063	−2.672	0.008
	LIPC -250G>A genotype	0.036	0.012	0.068	3.020	0.003
	LIPG 584C>T genotype	0.040	0.012	0.076	3.303	0.001
	LIPG 584C>T allele	0.039	0.014	0.064	2.781	0.005
	MTHFR 677C>T genotype	0.053	0.010	0.118	5.130	0.000
	MTHFR 677C>T allele	0.068	0.013	0.123	5.338	0.000
ApoB	MTHFR 677C>T genotype	0.032	0.008	0.086	3.781	0.000
	MTHFR 677C>T allele	0.042	0.010	0.092	4.049	0.000
ApoA1/ApoB	LIPC -250G>A genotype	0.070	0.026	0.063	2.649	0.008

TC, total cholesterol; TG, triglyceride; HDL-C, high-density lipoprotein cholesterol; LDL-C, low-density lipoprotein cholesterol; ApoA1, apolipoprotein A1; ApoB, apolipoprotein B; ApoA1/ApoB, the ratio of apolipoprotein A1 to apolipoprotein B; ABCA-1, ATP-binding cassette transporter A1; ACAT-1, acyl-CoA:cholesterol acyltransferase-1; LDL-R, low density lipoprotein receptor; LIPC, hepatic lipase gene; LIPG, endothelial lipase gene; MTHFR, methylenetetrahydrofolate reductase; MYLIP, the E3 ubiquitin ligase myosin regulatory light chain-interacting protein; PCSK9, proprotein convertase subtilisin-like kexin type 9; PPARD, peroxisome proliferator-activated receptor delta; SCARB1, Scavenger receptor class B type 1.

**Table 6:** Correlation between genotypes or alleles and serum lipid levels in the normal weight subjects

**Lipid**	**Genotype**/**allele**	**Unstandardized coefficient**	**Std. error**	**Standardized coefficient**	***t***	***P***
TC	ACAT-1 rs1044925 genotype	−0.131	0.053	−0.078	−2.477	0.013
	LDL-R *Ava*II genotype	−0.089	0.043	−0.062	−2.049	0.041
	LDL-R *Ava*II allele	−0.121	0.056	−0.066	−2.178	0.030
	LIPC -250G>A genotype	0.156	0.045	0.106	3.443	0.001
	LIPC -250G>A allele	0.204	0.057	0.111	3.593	0.000
	MTHFR 677C>T genotype	0.266	0.046	0.179	5.768	0.000
	MTHFR 677C>T allele	0.290	0.057	0.158	5.089	0.000
	PPARD +294T>C genotype	0.101	0.048	0.066	2.104	0.036
TG	LIPC -250G>A allele	0.149	0.065	0.072	2.292	0.022
	MYLIP rs3757354 genotype	−0.101	0.045	−0.071	−2.251	0.025
	SCARB1 rs5888 genotype	−0.142	0.066	−0.068	−2.157	0.031
HDL-C	ACAT-1 rs1044925 genotype	−0.061	0.028	−0.068	−2.177	0.030
	LIPC -250G>A genotype	0.045	0.022	0.062	2.040	0.042
	LIPG 584C>T genotype	0.085	0.025	0.104	3.364	0.001
	LIPG 584C>T allele	0.085	0.030	0.089	2.877	0.004
	MTHFR 677C>T genotype	0.105	0.024	0.134	4.361	0.000
	MTHFR 677C>T allele	0.129	0.030	0.134	4.352	0.000
	MYLIP rs3757354 genotype	0.054	0.021	0.082	2.609	0.009
LDL-C	LIPC -250G>A genotype	0.080	0.037	0.067	2.171	0.030
	LIPC -250G>A allele	0.125	0.046	0.084	2.713	0.007
	MTHFR 677C>T genotype	0.146	0.036	0.126	4.016	0.000
	MTHFR 677C>T allele	0.156	0.045	0.109	3.493	0.000
ApoA1	ACAT-1 rs1044925 genotype	−0.047	0.018	−0.080	−2.606	0.009
	LIPC -250G>A genotype	0.048	0.016	0.087	2.969	0.003
	LIPC -250G>A allele	0.051	0.020	0.074	2.516	0.012
	LIPG 584C>T genotype	0.040	0.016	0.075	2.467	0.014
	LIPG 584C>T allele	0.049	0.019	0.079	2.608	0.009
	MTHFR 677C>T genotype	0.057	0.014	0.121	3.965	0.000
	MTHFR 677C>T allele	0.071	0.018	0.122	4.019	0.000
	SCARB1 rs5888 genotype	−0.034	0.012	−0.065	−2.742	0.006
ApoB	LIPC -250G>A allele	0.033	0.013	0.078	2.498	0.013
	MTHFR 677C>T genotype	0.037	0.011	0.105	3.359	0.001
	MTHFR 677C>T allele	0.039	0.013	0.091	2.904	0.004
	SCARB1 rs5888 allele	−0.012	0.010	−0.048	−2.043	0.041

TC, total cholesterol; TG, triglyceride; HDL-C, high-density lipoprotein cholesterol; LDL-C, low-density lipoprotein cholesterol; ApoA1, apolipoprotein A1; ApoB, apolipoprotein B; ApoA1/ApoB, the ratio of apolipoprotein A1 to apolipoprotein B; ABCA-1, ATP-binding cassette transporter A1; ACAT-1, acyl-CoA:cholesterol acyltransferase-1; LDL-R, low density lipoprotein receptor; LIPC, hepatic lipase gene; LIPG, endothelial lipase gene; MTHFR, methylenetetrahydrofolate reductase; MYLIP, the E3 ubiquitin ligase myosin regulatory light chain-interacting protein; PCSK9, proprotein convertase subtilisin-like kexin type 9; PPARD, peroxisome proliferator-activated receptor delta; SCARB1, Scavenger receptor class B type 1.

**Table 7:** Correlation between genotypes or alleles and serum lipid levels in the overweight/obese subjects

**Lipid**	**Genotype/allele**	**Unstandardized coefficient**	**Std. cp error**	**Standardized coefficient**	***t***	***P***
TC	ABCA-1 V825I all`ele	0.162	0.078	0.074	2.078	0.038
	LIPG 584C>T genotype	0.228	0.073	0.109	3.122	0.002
	LIPG 584C>T allele	0.251	0.084	0.105	2.993	0.003
	MTHFR 677C>T genotype	0.158	0.060	0.092	2.624	0.009
	MTHFR 677C>T allele	0.245	0.075	0.115	3.267	0.001
	MYLIP rs3757354 genotype	−0.121	0.054	−0.082	−2.264	0.024
	PPARD +294T>C allele	0.222	0.083	0.096	2.666	0.008
	SCARB1 rs5888 allele	−0.182	0.069	−0.095	−2.633	0.009
TG	MTHFR 677C>T genotype	0.272	0.106	0.090	2.556	0.011
	PCSK9 E670G genotype	1.251	0.309	0.137	4.053	0.000
	PPARD +294T>C allele	0.675	0.179	0.133	3.763	0.000
	SCARB1 rs5888 genotype	0.232	0.099	0.084	2.337	0.020
HDL-C	ABCA-1 V825I genotype	−0.048	0.022	−0.077	−2.193	0.029
	LIPG 584C>T genotype	0.087	0.026	0.114	3.337	0.001
	LIPG 584C>T allele	0.068	0.030	0.078	2.270	0.024
	MTHFR 677C>T genotype	0.097	0.023	0.146	4.122	0.000
	MTHFR 677C>T allele	0.138	0.029	0.167	4.721	0.000
	PCSK9 E670G genotype	−0.167	0.075	−0.076	−2.234	0.026
	SCARB1 rs5888 genotype	−0.069	0.029	−0.084	−2.265	0.018
LDL-C	ABCA-1 V825I genotype	0.134	0.040	0.119	3.308	0.001
	ABCA-1 V825I allele	0.228	0.061	0.134	3.726	0.000
	LIPG 584C>T genotype	0.115	0.054	0.076	2.106	0.035
	LIPG 584C>T allele	0.131	0.062	0.077	2.116	0.035
ApoA1	ABCA-1 V825I allele	−0.044	0.020	−0.075	−2.138	0.033
	LIPG 584C>T genotype	0.036	0.018	0.071	2.038	0.042
	MTHFR 677C>T genotype	0.057	0.015	0.137	3.862	0.000
	MTHFR 677C>T allele	0.076	0.018	0.145	4.099	0.000
	SCARB1 rs5888 genotype	−0.062	0.018	−0.122	−3.523	0.000
ApoB	ABCA-1 V825I genotype	0.023	0.011	0.074	2.041	0.042
	ABCA-1 V825I allele	0.046	0.017	0.096	2.652	0.008
	LIPC -250G>A genotype	−0.034	0.012	−0.101	−2.777	0.006
	MTHFR 677C>T genotype	0.028	0.013	0.077	2.147	0.032
	MTHFR 677C>T allele	0.048	0.016	0.107	2.992	0.003
	MYLIP rs3757354 genotype	−0.026	0.012	−0.079	−2.190	0.029
	SCARB1 rs5888 allele	−0.052	0.015	−0.129	−3.587	0.000
ApoA1/ApoB	ABCA-1 V825I genotype	−0.093	0.029	−0.114	−3.168	0.002
	ABCA-1 V825I allele	−0.185	0.044	−0.149	−4.172	0.000
	LIPC -250G>A genotype	0.132	0.036	0.132	3.698	0.000
	LIPC -250G>A allele	0.114	0.048	0.085	2.361	0.018
	MYLIP rs3757354 genotype	0.058	0.030	0.071	1.965	0.050

TC, total cholesterol; TG, triglyceride; HDL-C, high-density lipoprotein cholesterol; LDL-C, low-density lipoprotein cholesterol; ApoA1, apolipoprotein A1; ApoB, apolipoprotein B; ApoA1/ApoB, the ratio of apolipoprotein A1 to apolipoprotein B; ABCA-1, ATP-binding cassette transporter A1; ACAT-1, acyl-CoA:cholesterol acyltransferase-1; LDL-R, low density lipoprotein receptor; LIPC, hepatic lipase gene; LIPG, endothelial lipase gene; MTHFR, methylenetetrahydrofolate reductase; MYLIP, the E3 ubiquitin ligase myosin regulatory light chain-interacting protein; PCSK9, proprotein convertase subtilisin-like kexin type 9; PPARD, peroxisome proliferator-activated receptor delta; SCARB1, Scavenger receptor class B type 1.

## Discussion

### Serum lipid levels in the overweight/obese subjects

In the present study, we showed that the levels of serum TC, TG, LDL-C, ApoA1, and ApoB were higher, and the levels of serum HDL-C and the ratio of ApoA1 to ApoB were lower in overweight/obese than in normal weight subjects. This is in agreement with those of previous studies [13-17]. Dyslipidemia in the obesity may be associated with insulin resistance [37-40]. Insulin is a lipid-synthetic hormone, thus alteration in a gene regulating insulin gene transcription may alter lipid metabolism as well and contribute to dyslipidemia. The liver is the main target organ of the insulin effect. Insulin resistance can descend the repression of insulin on the concentrations of plasma free fatty acids, increase the plasma levels of free fatty acids, promote free fatty acids into the liver, and stimulate the synthesis and release of very low density lipoprotein (VLDL) in the liver. At the same time, insulin resistances can also decline the activity of lipoprotein lipase (LPL), reduce the metabolism of VLDL, and increase the levels of plasma VLDL.

### Genotypic and allelic frequencies in different populations

We showed that the genotypic and allelic frequencies of LIPC -250G>A and PCSK9 E670G were different between normal weight and overweight/obese subjects, the overweight/obese subjects had higher LIPC -250A and PCSK9 670A allele frequencies than normal weight subjects. The genotypic frequency of LIPG 584C>T and allelic frequency of MYLIP rs3757354 were also different between normal weight and overweight/obese subjects. The allelic frequencies of LIPC -250G>A between African Americans and white Americans were quite different in several previous studies: the less common allele (−250A) of the LIPC polymorphisms in white Americans was the more common allele in African Americans [41,42]. The frequency of the LIPC -250A allele was found to range between 15-21% among Caucasians [41], 32% among Brazilian [43], 39% among Taiwanese-Chinese [44], 45-53% among African Americans [41,42] and 47% among Japanese-Americans [41]. The frequency of PCSK9 670G allele was rare in whites but present in approximately 24.8% of blacks [45]. Kotowski et al. [46] also reported that the minor-allele frequency (670G) in the Dallas Heart Study (DHS) was 3.6% in whites, 4.2% in Hispanics, and 26.0% in blacks. The frequency of the PCSK9 670G allele in patients selected from Universitätsklinikum Hamburg-Eppendorf Martinistrasse, Hamburg, Germany was 5% [47] which lies between that observed in the TexGen population, 4.4% and that reported for the Lipoprotein Coronary Atherosclerosis Study, 7.4% by Chen et al. [48] in their original study. There was no significant difference in the frequency of the PCSK9 670G allele in patients with LDL-C below the 50th percentile for age and sex, 4.4%, those with LDL-C between the 50th and 95th percentiles, 6.4% and those with LDL-C above the 95th percentile, 6.4% [47]. The 670G carrier in Chinese Taiwanese was identified less frequently in patients with CAD than in controls (9.9% vs. 11.9%), but the difference was not significant in a multivariable logistic regression analysis [49]. The frequency of LIPG 584T allele was 10.3% in blacks, 31.2% in white controls, 32.6% in whites with high HDL-C [50], 26% in the Lipoprotein and Coronary Atherosclerosis Study population (white individuals, but including 27 or 7% African Americans) [51], 26% in Japanese [52], and 21.6% in healthy school-aged Japanese children [53].

### Associations between SNPs and serum lipid levels

The potential associations between these lipid-related gene SNPs and serum lipid levels [27-35] or obesity [54-65] in humans have been evaluated in a large number of studies. However, previous findings on the association of these SNPs with the changes in serum lipid levels or obesity (BMI) are inconsistent in different racial/ethnic groups. ABCA1 R219K (rs2230806) was associated with altered plasma HDL-C concentrations. This association on HDL-C levels was modified by BMI in a Chinese population from Chengdu area [54]. ABCA1 gene variant (R230C, rs9282541) apparently exclusive to Native American individuals was also associated with low HDL-C levels, obesity and type 2 diabetes in Mexican Mestizos. The 230C allele was associated with lower HDL-C levels and with higher BMI in the combined analysis of Native American populations [55]. Kitjaroentham et al. [56] showed that overweight/obese men carrying the mutant allele of ABCA1 R219K had lower HDL-C levels than the controls. However, no positive association was observed using bivariate logistic regression analysis. On the contrary, there was no difference in HDL-C levels among genotypes in ABCA1 I883M (rs4149313) polymorphism. No difference was detected in genotypic frequency between the overweight/obese and control subjects for both polymorphisms. Two previous cross-sectional association studies showed that LDL-R *Apa*LI and *Hinc*II polymorphisms were associated with obesity in essential hypertensives but not in normotensives [57,58]. Griffiths et al. [59] showed that LDL-R microsatellite marker, located more towards the 3′ end of the gene, was associated with obesity in the normotensive population studied. There was also a significant association between variants of the LDL-R microsatellite and obesity, in the overall tested population, due to a contributing effect in females, but not in males [60]. These results indicate that LDL-R could play an important role in the development of obesity, which might be independent of hypertension or sex. MTHFR 677T allele was associated with obesity, hypertriglyceridemia and low HDL-C levels [61]. Birth weights were lower in 677TT than in 677CC and 677CT subjects, as well as birth lengths [62]. In subjects with and without type 2 diabetes, PPARD +294T>C was associated with BMI, HDL-C, leptin, and TNF-alpha and was dependent on gender [63]. BMI in metabolic syndrome patients with +294C allele carriers were significantly higher than that of TT genotype [64]. However, Aberle et al. [65] showed that the PPARD +294C allele was significantly associated with a lower BMI. In this study, we showed that the levels of TC, ApoA1 (ABCA-1); TC, LDL-C, ApoA1, ApoB and ApoA1/ApoB (LIPC); TG, HDL-C, and ApoA1 (LIPG); TC, HDL-C, LDL-C, ApoA1 and ApoB (MTHFR); HDL-C and ApoA1 (MYLIP) in normal weight subjects were different among the genotypes. The levels of LDL-C, ApoB and ApoA1/ApoB (ABCA-1); HDL-C, ApoA1, ApoB and ApoA1/ApoB (LIPC); TC, HDL-C, ApoA1 and ApoB (LIPG); TC, TG, HDL-C, LDL-C, ApoA1 and ApoB (MTHFR); TC, TG and ApoB (MYLIP); TG (PCSK9); TG and ApoA1 and ApoB (PPARD); and TC, HDL-C, LDL-C, ApoA1 and ApoB (SCARB1) in overweight/obese subjects were also different among the genotypes. Serum lipid levels were also associated with the genotypes or alleles of several SNPs in the combined population of normal weight and overweight/obese subjects, normal weight subjects, and overweight/obese subjects; respectively. These results suggest that the associations of these SNPs and serum lipid levels are different between normal weight and overweight/obese subjects.

### Interactions between SNPs and overweight/obesity on serum lipid levels

The interactions of SNPs and overweight/obesity on serum lipid parameters are limited. In an examination of the effect of body fat on the genotypic effects in the children, Talmud et al. [66] showed that there was significant interaction between tertiles of sum of skinfold thickness and the LIPC -480C>T genotype in determining HDL-C levels. In the lowest tertile, carriers of the -480T allele had lower mean HDL-C levels, whereas in the two upper tertiles, -480T carriers had higher mean HDL-C levels when compared with the CC homozygotes. In a previous study [67], the interaction between BMI, the LIPC -514C>T polymorphism, and hepatic lipase activity was examined in white and African American men. The results showed that increased BMI was associated with increased hepatic lipase activity in men. A striking additive effect of BMI and the LIPC -514C>T polymorphism on hepatic lipase activity was observed. The joint effects of BMI and LIPC -514C>T genotypes strongly influenced hepatic lipase activity and were significantly greater than the effects of either factor considered alone. LIPC -514C>T and LIPC -250G>A SNPs have been demonstrated in complete linkage disequilibrium [30]. There was evidence for an interaction of the LIPC promoter polymorphism with visceral obesity in determining the level of hepatic lipase activity, the presence of the LIPC -514T allele seems to attenuate the increase in hepatic lipase activity with high levels of intra-abdominal fat [68]. The interactions of LIPC -514C>T and LIPC -250G>A SNPs and obesity on HDL-C levels were also observed in Taiwanese-Chinese men but not in women [44]. For males, significant interactions were noted between the two studied polymorphisms and obesity on HDL-C levels. For obese males, significantly higher HDL-C levels were found for carriers of the -514T and -250A alleles in comparison with non-carriers. In contrast, no significant differences were found for non-obese male subjects bearing different genotypes of the LIPC -514C>T and -250G>A polymorphisms. For both obese women and non-obese ones, no significant difference was detected between different genotypes of the two studied polymorphisms and HDL-C levels [44]. The beneficial effect of the LIPC -514T allele on plasma HDL_2_-C levels was abolished in the presence of visceral obesity [69]. Swarbrick et al. [70] reported that obese subjects carrying the Ala allele of the Pro12Ala polymorphism of the PPAR-gamma2 gene, but not the non-obese analogs, had a greater risk of developing combined hyperlipidemia and low-HDL-C levels. In the current study, we provide important insights for the interactions of several SNPs and overweight/obesity on serum lipid phenotypes. The SNPs of ABCA-1 (LDL-C and ApoA1/ApoB); LIPC (TC, LDL-C, ApoA1 and ApoB); LIPG (ApoB); MTHFR (TC, TG and LDL-C); MYLIP (TC and TG); PCSK9 (TG, HDL-C, ApoB and ApoA1/ApoB); PPARD (TG and ApoA1/ApoB); and SCARB1 (TG, ApoA1 and ApoB) interacted with overweight/obesity to modulate serum lipid levels. These findings suggest that some serum lipid parameters in our study subjects were partly influenced by the interactions of several SNPs and overweight/obesity. To the best of our knowledge, the interactions of ABCA-1, LIPG, MTHFR, MYLIP, PCSK9, PPARD, and SCARB1 SNPs and overweight/obesity on serum lipid parameters have not been previously explored.

### Study limitations

This study has several limitations. First, the levels of education, weight, systolic blood pressure, diastolic blood pressure, and the percentages of subjects who consumed alcohol were higher in overweight/obese than in normal weight subjects, whereas the percentages of subjects who smoked cigarettes were lower in overweight/obese than in normal weight subjects. Although sex, age, education level, physical activity, blood pressure, alcohol consumption, and cigarette smoking have been adjusted for the statistical analysis, we could not completely eliminate the potential effects of these factors on serum lipid levels among different genotypes in both groups. Second, the diet was not adjusted for the statistical analysis. In the present study, however, the diet in this isolated population is consistent throughout the year and among individuals because of the Bai Ku Yao's reliance on a limited number of locally available food items. Their staple food is corn gruel or corn tortillas. On ordinary days, they are vegetarians [7-9]. Finally, it is clearly established that serum lipid levels are regulated by multiple environmental and genetic factors, and their interactions [7-12]. Although we have detected the interactions of ten SNPs and overweight/obesity on serum lipid levels in this study, there are still many unmeasured environmental and genetic factors and their interactions. Thus, the interactions of gene-gene, gene-environment, and environment-environment on serum lipid levels remain to be determined. As a matter of fact, the interrelationship of SNPs and overweight/obesity on serum lipid levels is extremely complex, and was overlooked. For example, obesity-related genes play a role in the central regulation of energy balance, but some of the genes have also a role in the adipose tissue itself [71]. Uncoupling protein 2 gene polymorphisms are associated with obesity in some Asian populations [72]. Reduction in serum IL-18 levels across increasing numbers of +183 G-alleles (rs 5744292) is especially apparent in patient with diabetes type 2 and metabolic syndrome, suggesting a beneficial GG genotype in relation to cardiovascular outcome in these patients [73]. The impact of proteasome modulator 9 (PSMD9) gene within the chromosome 12q24 locus on hypercholesterolemia and contribution to cardio- and cerebrovascular events and inflammation may be high [74,75].

## Conclusion

Several SNPs in normal weight and overweight/obese subjects were found to be associated with serum lipid levels in the Guangxi Bai Ku Yao population. The interactions of ABCA-1, LIPC, LIPG, MTHFR, MYLIP, PCSK9, PPARD, and SCARB1 genotypes and overweight/obesity on serum lipid levels were detected. These results suggest that the differences in serum lipid levels between normal weight and overweight/obese subjects might partly result from different interactions of several SNPs and overweight/obesity. The observed associations and interactions between these SNPs and serum lipid parameters in this isolated ethnic subgroup may also be the major characteristics of these conditions in the other ethnic groups, especially in the minorities. However, large studies of populations with different ethnic origins are required to confirm these observations.

## Abbreviations

ABCA-1: ATP-binding cassette transporter A1; ACAT-1: Acyl-CoA:cholesterol acyltransferase-1; ANCOVA: Analysis of covariance; Apo: Apolipoprotein; BMI: Body mass index; CAD: Coronary artery disease; DNA: Deoxyribonucleic acid; HDL-C: High-density lipoprotein cholesterol; LDL-C: Low-density lipoprotein cholesterol; LDL-R: Low density lipoprotein receptor; LIPC: Hepatic lipase gene; LIPG: Endothelial lipase gene; LPL: Lipoprotein lipase; MTHFR: Methylenetetrahydrofolate reductase; MYLIP: The E3 ubiquitin ligase myosin regulatory light chain-interacting proteinPCR, Polymerase chain reaction; PCSK9: Proprotein convertase subtilisin-like kexin type 9; PPARD: Peroxisome proliferator-activated receptor delta; RFLP: Restriction fragment length polymorphism; SCARB1: Scavenger receptor class B type 1; SNPs: Single nucleotide polymorphisms; TC: Total cholesterol; TG: Triglyceride; VLDL: Very low density lipoprotein; WHO: World Health Organization.

## Competing interests

The authors declare that they have no competing interests.

## Authors' contributions

RXY conceived the study, participated in the design, carried out the epidemiological survey, collected the samples, performed statistical analyses, and drafted the manuscript. DFW, LM, LHHA, XLC, TTY, XJL, WYL, LZ, and ML participated epidemiological survey and undertook genotyping. DFW also helped to perform statistical analyses. All authors read and approved the final manuscript.
